# Editorial: SARS-CoV-2: From Genetic Variability to Vaccine Design

**DOI:** 10.3389/fgene.2022.960107

**Published:** 2022-08-26

**Authors:** Indrajit Saha, Nimisha Ghosh, Dariusz Plewczynski

**Affiliations:** ^1^ Department of Computer Science and Engineering, National Institute of Technical Teachers’ Training and Research, Kolkata, India; ^2^ Department of Computer Science and Information Technology, Institute of Technical Education and Research, Siksha ‘O’ Anusandhan (Deemed to be University), Bhubaneswar, India; ^3^ Faculty of Mathematics, Informatics and Mechanics, University of Warsaw, Warsaw, Poland; ^4^ Laboratory of Bioinformatics and Computational Genomics, Faculty of Mathematics and Information Science, Warsaw University of Technology, Warsaw, Poland; ^5^ Laboratory of Functional and Structural Genomics, Centre of New Technologies, University of Warsaw, Warsaw, Poland

**Keywords:** correlation, COVID-19, mutations, prediction, SARS-CoV-2, vaccine

## 1 Introduction

The whole world has been at a standstill for more than 2 years now due to the pandemic of COVID-19, the disease caused by the SARS-CoV-2 virus. The first case of COVID-19 was detected in Wuhan, China in December 2019, and the rest, as they say, is history. The disease has claimed more than 6 million lives worldwide. SARS-CoV-2 is a positive-stranded RNA virus with a length of about 30 kb encompassing non-structural and structural proteins. Spike glycoprotein, a structural protein present on the virus surface plays an important role in binding with human ACE2 and other receptors. Since its detection in Wuhan, the virus has mutated several times and has given way to variants such as B.1.1.7 (Alpha), B.1.351 (Beta), B.1.525 (Eta), B.1.427/B.1.429 (Epsilon), B.1.526 (Iota), B.1.617.1 (Kappa), B.1.617.2 (Delta), C.37 (Lambda), P.1 (Gamma), P.2 (Zeta), P.3 (Theta), and B.1.1.529 (Omicron).

In the initial days of the pandemic, there was little to no knowledge of this deadly virus. Thus, to understand the virus, whole genome analysis, and viral protein-based comparisons were carried out which concluded that SARS-CoV-2 is mostly related to bat SARS-like coronaviruses. Though there have been viruses like SARS-CoV-1 and MERS-CoV which belong to the same family of Coronaviridae just like SARS-CoV-2, outbreaks were sporadic and they did not cause global pandemics. Moreover, since the virus shared similarities with other viruses, its prediction was yet another challenge that the research community faced. Also, phylogenetic analyses were carried out by different researchers around the world to understand the virus mutations which mostly take place in the Spike glycoprotein. In fact, tools like Nextstrain have been used to visualize the virus evolution as well. These efforts by the researchers helped in a lot of ways to understand the virus’s spread and its mutations. However, the studies are mostly focused on the structural proteins, especially Spike glycoprotein of SARS-CoV-2 while research on non-structural proteins is still underway. Such proteins can be investigated further to understand the virus and its mutations better.

The efforts of the researchers have also paved the way for the development of vaccines to fight against this deadly virus. There are several vaccines like Oxford-AstraZeneca, Pfizer-BioNTech, Moderna, Novavax, Covaxin, Sputnik V, and Johnson & Johnson which have been developed to date by scientists around the world. However, the developed vaccines are primarily designed to generate neutralizing antibodies against Spike glycoprotein. Moreover, due to the waning antibody response and some emerging variants like Omicron being somewhat resistant to the antibody response evoked by these vaccines, the long-term sustainability of these vaccines is a bit questionable. In this regard, T-cell responses against coronaviruses can last for a very long time which has been demonstrated by SARS and MERS viruses as well. All these factors have motivated us to have an issue on “SARS-CoV-2: From Genetic Variability to Vaccine Design” to benefit the scientific community. The articles covered in this issue are discussed in the subsequent section.

## 2 Research Topic Organization

This Research Topic is divided into three main sections: two papers discuss the prediction of the SARS-CoV-2 virus, four papers cover the virus mutations, and six papers discuss the various vaccines and therapeutics for COVID-19.

In the first part, we have focussed on the prediction of the virus by using machine learning and deep learning techniques. We believe that this section will appeal to researchers working in the field of artificial intelligence. This section is especially interesting as while one paper has worked to predict SARS-CoV-2 by using genomic information, the other one has used machine learning to reveal pathological factors for diseases associated with airway smooth muscle inflammation on multi-omics levels.

The second part encompasses the mutations in the virus. Understanding the virus mutation is very important as the mutations lead to the various variants of the virus. The works in this part mostly deal with multiple sequence alignment (MSA) to reveal the virus mutations. What makes this section non-trivial is the fact that MSA has been performed with a huge number of SARS-CoV-2 sequences by all the contributions.

The third and final section discusses the various vaccines and therapeutics that can be used to fight against SARS-CoV-2. As discussed earlier, though there are several vaccines already approved by different medical agencies, their sustainability is not known till now. Thus, apart from the vaccine host immune system modulation can also be considered to find alternative solutions. Also, epitope-based vaccines and other therapeutics can be taken into account. Furthermore, vaccination, COVID-19 incidence, and mortality have also been explored in one work in this section. Moreover, Spike glycoprotein and ORF8 protein of SARS-CoV-2 are also analyzed to provide clinical and therapeutic implications.

### 2.1 Prediction

In Saha et al., deep learning based predictor viz. COVID-DeepPredictor has been proposed to predict unknown sequences of SARS-CoV-2 as well as other pathogens like SARS-CoV-1, MERS-CoV, Ebola, Dengue, and Influenza. COVID-DeepPredictor uses Long Short Term Memory as Recurrent Neural Network where *k*-mer technique is used to generate Bag-of-Unique-Descriptors. COVID-DeepPredictor achieves 100% prediction accuracy on validation datasets while on test datasets, the accuracy is as high as 99%.

Zhang et al. explore SARS-CoV-2 infection in airway smooth muscles which may play an important role in several other inflammatory diseases as well. They have used machine-learning-based computational approaches to identify specific regulatory factors that contribute to the activation and simulation of airway smooth muscles. This will lead to the identification of potential regulatory mechanisms linking airway smooth muscle tissues and inflammatory factors which will eventually help in identifying specific pathological factors for diseases associated with airway smooth muscle inflammation on multi-omics levels.

### 2.2 Mutation

In Lin et al., multiple sequence alignment using a conserved sequence search algorithm has been optimized to align 24,768 sequences from the GISAID dataset. This will help in conserved sequence searches to segment long sequences as well as make large-scale multisequence alignment possible, thereby facilitating comprehensive gene mutation analysis.

In Saha et al., multiple sequence alignment of 71,038 SARS-CoV-2 genomes from 98 countries have been performed to identify hotspot mutations in SARS-CoV-2. This has led to the identification of 45 unique hotspot mutations. Such mutations include L452R, T478K, E484Q, and N501Y.

In Biswas et al., database DbNSP InC has been reported which provides information on the NSPs of SARS-CoV-2 extracted from patients in India. It provides functional information, mutations observed in samples of Indian patients, primary and secondary structural analyses, strain and mutation analyses as well as mutations observed in the deceased, mild, and asymptomatic patients samples along with the distribution of mutations across different Indian states and phylogenetic analysis.

In Cueno et al., the authors have generated spike models of endemic HCoVs (HCoV 229E, HCoV OC43, HCoV NL63, HCoV HKU1, SARS CoV, MERS CoV), original SARS-CoV-2 and variants of concern (Alpha, Beta, Gamma, and Delta). They propose that structural similarities among the pathogens may help ascertain immune cross-reactivity while differences may result in viral infection.

### 2.3 Vaccine and Therapeutics

In Majumdar et al., the differences in COVID-19 death and infection ratio between the urban and rural population in India have been explored to discuss the role of the immune system, comorbidities, and associated nutritional status that may play role in the death rate of COVID-19 patients in such populations. Furthermore, they have also focussed on strategies for developing masks, vaccines, and other diagnostics to combat COVID-19.

In Mazzocco et al., a novel *in-silico* approach based on artificial intelligence and bioinformatics methods have been put forth to support the design of epitope-based vaccines. Their methods have also been evaluated for predicting the immunogenicity of epitopes. They have also discussed the potential applicability of such epitopes for the development of a vaccine eliciting cellular immunity for COVID-19.

In Somogyi et al., selection of immunoprevalent SARS-CoV-2-derived T cell epitopes using an *in-silico* cohort of HLA-genotyped individuals with different ethnicities has been considered. The results of this work are significant for the development of highly efficient epitope-based vaccines against various pathogens and diseases as well.

In Valcarcel et al., the focus is on analyzing structural similarities of ORF8 protein of SARS-CoV-2 with immunological molecules such as IL-1, thereby contributing to the immunological deregulation observed in COVID-19.

In Fukutani et al., the association between vaccine implementations, the occurrence of new cases, and mortality rate have been tracked. They have used CaVaCo (Cases, Vaccinations, and COVID-19) tool to retrieve the COVID-19 cases as well as the deaths and vaccination data to compare and correlate vaccination coverage of the countries with other parameters.

In Bensussen et al., a minimal mathematical model of the effect of the extra copy of TMPRSS2 on ORF8 production and persistence in the infected cells of a Down syndrome patient having COVID-19 disease has been proposed. Their results support the hypothesis that people with Down syndrome have a high susceptibility to COVID-19 due to the overproduction of TMPRSS2.

